# A Multisubcellular Compartment Model of AMPA Receptor Trafficking for Neuromodulation of Hebbian Synaptic Plasticity

**DOI:** 10.3389/fnsyn.2021.703621

**Published:** 2021-08-11

**Authors:** Stefan Mihalas, Alvaro Ardiles, Kaiwen He, Adrian Palacios, Alfredo Kirkwood

**Affiliations:** ^1^Allen Institute for Brain Science, Seattle, WA, United States; ^2^Centro Interdisciplinario de Neurociencia de Valparaíso, Facultad de Ciencias, Universidad de Valparaíso, Valparaíso, Chile; ^3^Centro de Neurología Traslacional, Facultad de Medicina, Universidad de Valparaíso, Valparaíso, Chile; ^4^Mind Brain Institute, Johns Hopkins University, Baltimore, MD, United States

**Keywords:** G-protein coupled receptor, pull-push, long-term potentiation, long-term depression, cortex

## Abstract

Neuromodulation can profoundly impact the gain and polarity of postsynaptic changes in Hebbian synaptic plasticity. An emerging pattern observed in multiple central synapses is a pull–push type of control in which activation of receptors coupled to the G-protein Gs promote long-term potentiation (LTP) at the expense of long-term depression (LTD), whereas receptors coupled to Gq promote LTD at the expense of LTP. Notably, coactivation of both Gs- and Gq-coupled receptors enhances the gain of both LTP and LTD. To account for these observations, we propose a simple kinetic model in which AMPA receptors (AMPARs) are trafficked between multiple subcompartments in and around the postsynaptic spine. In the model AMPARs in the postsynaptic density compartment (PSD) are the primary contributors to synaptic conductance. During LTP induction, AMPARs are trafficked to the PSD primarily from a relatively small perisynaptic (peri-PSD) compartment. Gs-coupled receptors promote LTP by replenishing peri-PSD through increased AMPAR exocytosis from a pool of endocytic AMPAR. During LTD induction AMPARs are trafficked in the reverse direction, from the PSD to the peri-PSD compartment, and Gq-coupled receptors promote LTD by clearing the peri-PSD compartment through increased AMPAR endocytosis. We claim that the model not only captures essential features of the pull–push neuromodulation of synaptic plasticity, but it is also consistent with other actions of neuromodulators observed in slice experiments and is compatible with the current understanding of AMPAR trafficking.

## Introduction

Organisms learn about their environment from experiences that are rewarding, aversive, or salient. At an elementary level, learning is thought to result from changes in the strength of specific synaptic connections, changes which in most cases are determined by local patterns of neural activity in a Hebbian manner, which is long-term potentiation (LTP) when pre-synaptic and post-synaptic activities correlate; and long-term depression (LTD) when they do not correlate (Malenka and Nicoll, 1999; Malenka and Bear, 2004). These local synaptic changes, in turn, are subordinated to global behavioral states somehow via the action of the long-range and diffusely projecting monoaminergic and cholinergic neuromodulatory systems. Hence, understanding the neuromodulation of Hebbian plasticity is central to understanding the mechanisms of learning.

Neuromodulators can activate multiple G-protein coupled receptors (GPCRs) to affect Hebbian plasticity in multiple ways. Hebbian plasticity is initiated by the intracellular Ca^2+^ signal that ensues the activation of postsynaptic NMDA-receptors and/or metabotropic glutamate receptors (mGluRs) and voltage-gated Ca^2+^channels. When the magnitude of this Ca^2+^signal exceeds a certain LTD-threshold, it selectively activates phosphatases that promote the removal of AMPA receptors (AMPAR) out of the synapse, and when it exceeds a larger LTP-threshold the Ca^2+^ signal promotes the activation of kinases and the incorporation of AMPARs into the synapse (Malenka and Nicoll, 1999; Shouval et al., 2002; Malenka and Bear, 2004). A wealth of studies has reported that neuromodulators affect this Ca^2+^ signal directly by acting on NMDARs for example, or indirectly by modulating cellular and/or circuit excitability (Faber et al., 2008; Pawlak et al., 2010; Tritsch and Sabatini, 2012; Edelmann and Lessmann, 2013; Meunier et al., 2017; Bari et al., 2020; Fernandez de Sevilla et al., 2021; Lutzu and Castillo, 2021). On the other hand, multiple mechanisms for the expression of NMDA-dependent Hebbian plasticity have been identified. These include the direct exchange of AMPAR between the synapse and internal compartments via exocytosis in LTP, for example (Lledo et al., 1998; Ahmad et al., 2012; Wu et al., 2017), changes in AMPAR unitary conductance (Park et al., 2021), and lateral diffusion of surface AMPARs and their trapping at postsynaptic density compartment (PSD) site, in case of LTP, and their release from the PSD, in the case of LTD (Oh et al., 2006; Derkach et al., 2007; Makino and Malinow, 2009; Newpher and Ehlers, 2009; Choquet, 2018; Diering and Huganir, 2018). These are complex processes that involve AMPAR phosphorylation at specific sites, interactions with multiple synaptic proteins, and possibly transient insertion of calcium-permeable AMPARs (Nicoll, 2017; Buonarati et al., 2019; Purkey and Dell'Acqua, 2020). The recruitment of these mechanisms in different synapses likely varies depending on experimental conditions like the induction protocols used. Hence, like the role of transient insertion of calcium-permeable AMPARs, the contribution of these mechanisms to LTP/D expression is still under debate. In consequence, although neuromodulation also occurs at this stage (Huang et al., 2012), the exact mechanisms are less understood than in the case of neuromodulation of the induction of plasticity.

Despite the diverse receptor targets of neuromodulators, results from several studies are roughly consistent with a simple rule, which is the pull–push regulation of LTP and LTD by receptors coupled to the G-proteins Gs and Gq. According to this rule, Gs-coupled receptors like the D1- dopaminergic receptor or the β-adrenoreceptor, which stimulate cAMP production, tend to promote LTP, but often at the expense of LTD (Thomas et al., 1996; Katsuki et al., 1997; Mockett et al., 2007; Seol et al., 2007; Lin et al., 2008; Huang et al., 2012; Nguyen and Gelinas, 2018; Brzosko et al., 2019). Conversely, Gq-coupled receptors that stimulate the phospholipase C cascade, like the α1-adrenoreceptor or the M1 cholinergic receptor, tend to promote LTD at the expense of LTP (Choi et al., 2005; Seol et al., 2007; Takamatsu et al., 2008; Huang et al., 2012; Hulme et al., 2012). In a previous study examining this pull–push regulation, we showed that it occurs at the level of the expression of plasticity. We also observed that while Gs-coupled receptors inhibit LTD and Gq-coupled receptors inhibit LTP when stimulated individually, and when acting together, they enhance the gain of both LTP and LTD (Seol et al., 2007; Huang et al., 2012). To account for these intriguing interactions between GPCRs, we propose a simple kinetic model in which AMPAR are trafficked in and out of the synapse through perisynaptic (peri-PSD) compartments of limited size (which models the presence of a limited amount of structural anchoring proteins). In the model, the expression of LTP and LTD is limited by the occupancy of AMPARs at the small perisynaptic compartments. GPCRs, in turn, regulate the LTP/D expression by controlling the filling of these perisynaptic compartments. We surmise that the described model captures several essential features of the data observed in the visual cortex and its application could be extended to neuromodulation in other structures.

## Results

**Figure 1:**
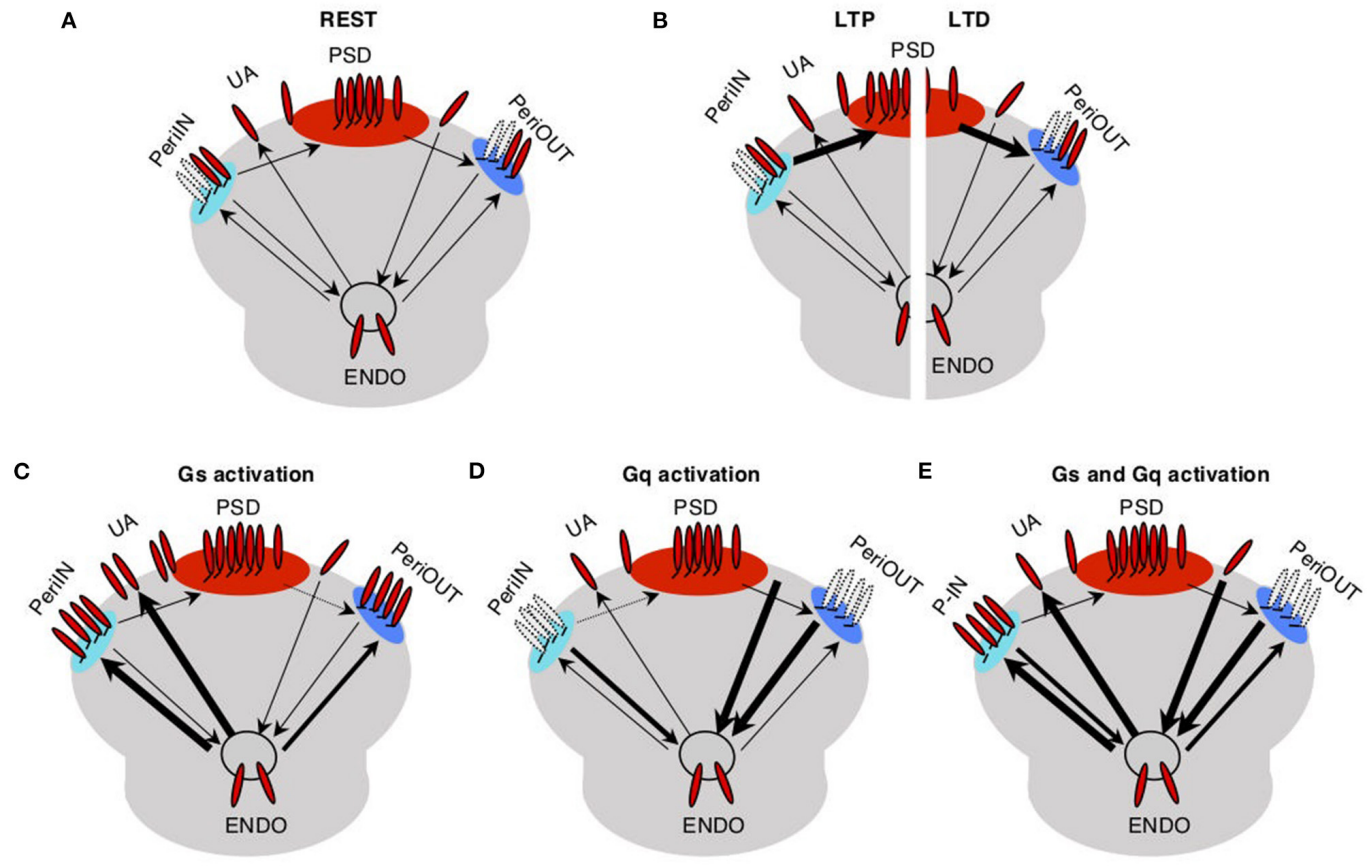
Trafficking model of neuromodulation of Hebbian plasticity. AMPA receptors (red) at the synapse are either anchored at the postsynaptic density compartment (PSD) and in two close compartments (PeriIN, PeriOut), or unanchored. **(A)** Traffic between these synaptic compartments and with an endoplasmatic compartment (Endo) is low at rest (thin arrows). **(B)** Traffic between PSD and P-In, P-Out increases during long-term potentiation (LTP; left) and long-term depression (LTD; right) induction (thick arrows). **(C)** Activation of Gs increases traffic out of the Endo toward the synapse saturating both P-In and P-Out, thus preventing LTD. **(D)** Activation of Gq increases traffic out of the synapse toward the Endo compartment depleting both P-In and P-Out, thus preventing LTP. **(E)** Activation of both Gs and Gq increases the exchange between the synaptic and the Endo compartments, thus enabling/facilitating both, LTP and LTD.

The trafficking model for the pull–push neuromodulation of Hebbian plasticity is illustrated in Figure 1. Essentially, it involves AMPAR trafficking between four saturable membrane compartments and one non-saturable internal endocytic compartment (endo). AMPARs in the PSD contribute to the synaptic conductance, and they can be trafficked to and from small peri-PSD compartments (PeriIN, PeriOut), which do not contribute to the synaptic responses. The model also features the neuromodulation of a direct exchange between the endocytic compartment and unanchored (UA) freely moving AMPARs, a fraction of which contribute to synaptic responses. This was a necessary minimal assumption to model the conspicuous, yet transient changes in synaptic responses induced by GPCR agonists alone (Huang et al., 2012).

We assume that at rest traffic between all the compartments is slow. LTP induction temporarily and selectively increases the traffic rate of AMPARs from the PeriIN to the PSD compartment, thus increasing synaptic conductance (Figure 1B left). Conversely, LTD induction temporarily increases the rate of traffic out of the PSD to empty slots in the PeriOut compartment, thus reducing synaptic conductance (Figure 1B right). We also assume that the PeriIN and PeriOut compartments have limited capacity. Consequently, LTP and LTD are constrained by the occupancy of these compartments. LTP would be limited by the number of AMPARs anchored at PeriIN and LTD by the number of empty slots at PeriOut.

The key feature of the model is that GPCRs control the expression of LTP and LTD by determining the occupancy of the PeriIN and PeriOut compartments. Activation of GPCRs increases AMPAR traffic of the endocytic compartment with the two perisynaptic compartments and with the UA pool of AMPARs. Gs-coupled receptors increase exocytosis to the PeriIN, the UA AMPARs, and to a lesser extent the PeriOut compartments (Figure 1C). Gq-coupled receptors increase the endocytosis from the PeriOut, the UA AMPARs, and to a lesser extent the PeriIN compartments (Figure 1D). Thus, activation of Gs-coupled receptors would prevent LTD by saturating the PeriOut compartment (Figure 1C), whereas Gq activation would deplete the PeriIN compartment and prevent LTP (Figure 1D). The coactivation of Gs- and Gq-coupled receptors would saturate the PeriIN and deplete the PeriOut compartments because of their differential effects on the exchange between the endo and perisynaptic compartments, thus enabling the expression of both LTP and LTD.

The central motivation for the trafficking model outlined above was a study that examined in slices how agonists for β- and α-adrenergic receptors (respectively, coupled to Gs and Gq) affect LTP and LTD induced by pairing conditioning. The study showed that, indeed, activation of β-adrenoreceptors promotes LTP at the expense of LTD, activation of α-adrenoreceptors promotes LTP at the expense of LTD, and, importantly, activation of both together (β- and α-adrenoreceptors) promote both LTP and LTD. We asked, therefore, whether with reasonable assumptions, the trafficking could account for that experimental data on neuromodulation of LTP and LTD. The equations governing the AMPAR trafficking between compartments used in the fitting and the values of the constants and parameters are detailed in the Materials and Methods section. Briefly, the rates of trafficking to and from the PSD compartments during LTP and LTD were assumed to depend on kinases and phosphatases activated by Ca influx during the pairing conditioning, whereas the movement of unanchored AMPARs was assumed limited by lateral diffusion.

**Figure 2:**
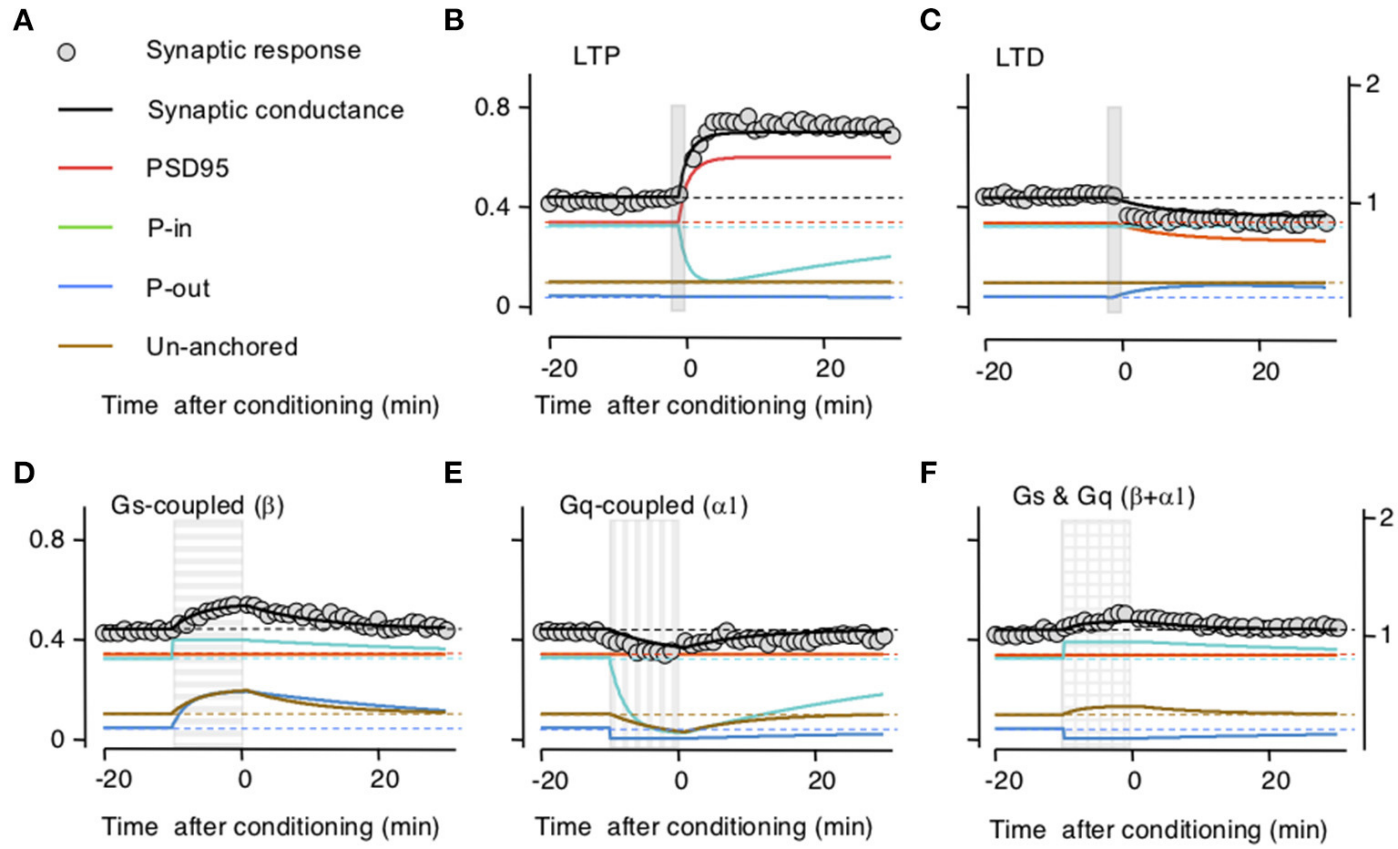
Simulation of how long-term potentiation (LTP)/long-term depression (LTD) induction and the stimulation of Gs and Gq-coupled receptors change the occupancy of the modeled AMPA receptor (AMPAR) compartments. **(A)** Color conventions of the various synaptic compartment described in the remaining panels. Also included are gray circles representing actual synaptic response data from Huang et al. (2012). **(B)** An LTP-induction protocol (vertical gray bar) transiently increases AMPAR traffic onto the postsynaptic density compartment (PSD)95 from the PeriIN compartment, depleting it, but without affecting the PeriOut compartment. This results in a net increase in synaptic conductance with a time course, comparable with changes reported in Huang et al. (2012). Left *Y*-axis: occupancy of synaptic compartments; right *Y*-axis: normalized synaptic response. **(C)** An LTD protocol (vertical gray bar) transiently increases traffic out of the PSP onto the PeriOut compartment, filling it up, and reducing synaptic conductance. **(D)** Stimulation of Gs-coupled receptors (horizontally striped bar) increases AMPAR trafficking from the endosomatic compartment into all but the PSD synaptic compartments causing a transient increase in synaptic conductance. **(E)** Stimulation of Gq-coupled receptors (vertically striped bar) increases AMPAR trafficking to the endosomatic compartment from all but the P synaptic compartments causing a transient decrease in synaptic conductance compartments. **(F)** Stimulation of Gs-and Gq-coupled receptors (checkered bar) increases AMPAR trafficking (in and out) between the endosomal and all but the PDS95 synaptic compartments. This fills up the PeriIN compartment, depletes the PeriOut compartment, and results in a modest transient increase in synaptic conductance.

To test the model, we first optimized parameters to fit the time course and magnitude of the reported changes in synaptic responses after LTP or LTD induction and after activation of β-and/or α-adrenoreceptors separately. Then we tested the ability of the model to reproduce the interactions between LTP/D and β-and/or α-adrenoreceptors. In the experimental study, the noradrenergic agonists were applied for 10 min, whereas LTP/D were induced with a 2-min pairing of synaptic activation with postsynaptic depolarization delivered by the end of the agonist application (Huang et al., 2012). Parameters optimized included the synaptic size of the compartment, the exchange rates after LTP/D, and the GPCRs activation. The optimization aimed to fit the results reported in Figure 1 of Huang et al. (2012), which capture the essence of the pull–push nature of the neuromodulation of LTP/D. As shown in Figure 2, with adequate parameters and initial values, the changes in synaptic conductance calculated with the model (thick black lines) fit the experimental data (gray circles) in each of the five conditions. Note that in the model LTP and β-adrenoreceptor activation, both potentiate the synaptic response, but through different mechanisms (increasing AMPAR at the PSD or the UA ones) and with opposite effects on the PeriIN compartments. LTP depletes the PeriIN compartment whereas the β-adrenoreceptor saturates it (Figures 2B,D). Conversely, LTD and α-adrenoreceptor activation both depress the synaptic response by reducing PSD-anchored and UA AMPARs, respectively, but with opposite effects on the occupancy of the PeriOut compartment (Figures 2C,E). On the other hand, coactivation of β- and α-adrenoreceptors modestly affect the synaptic responses, as their effects on the unanchored AMPAR pool cancel out; yet they saturate the PeriIN and deplete the PeriOut compartments (Figure 2F).

**Figure 3:**
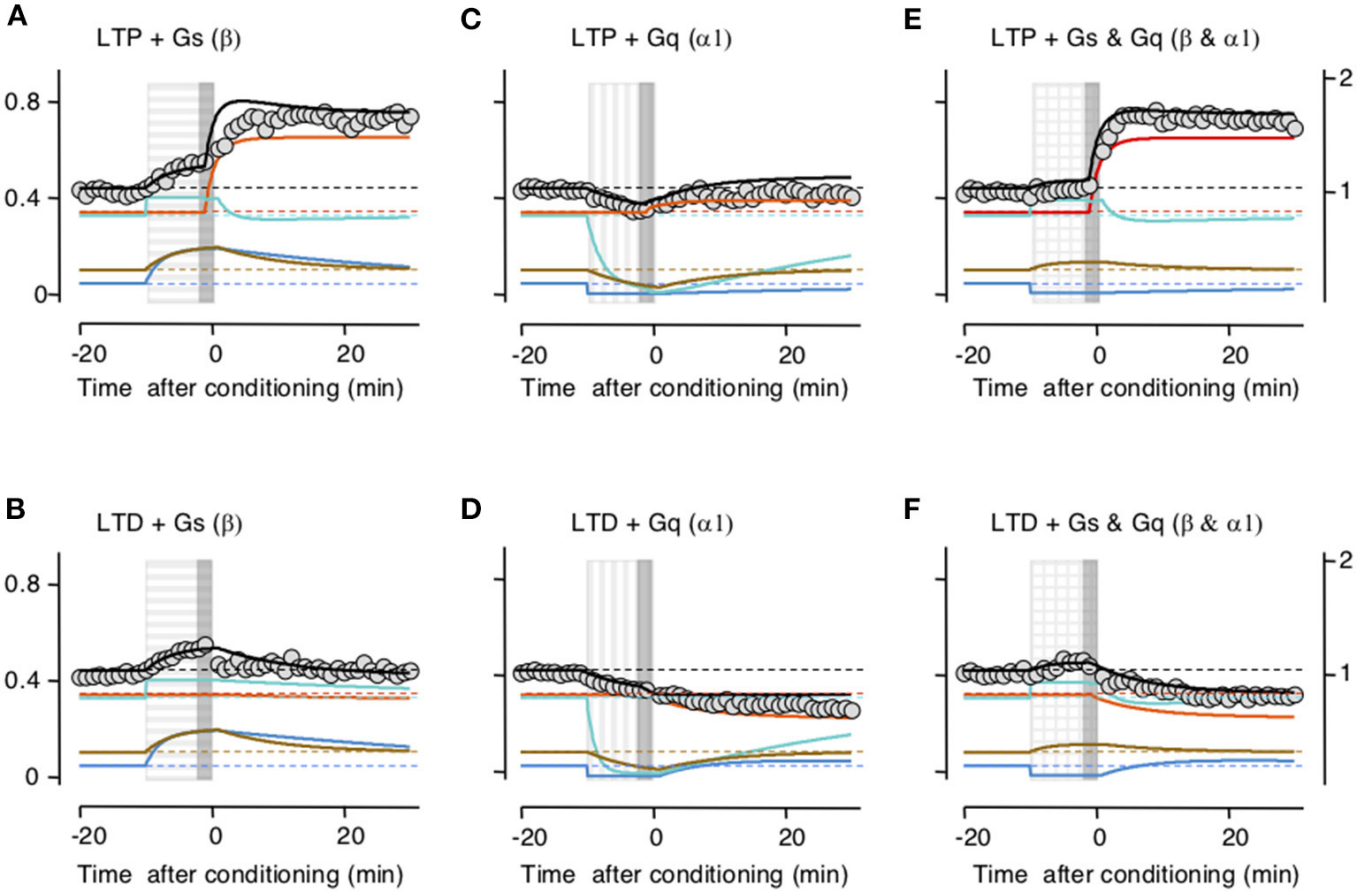
Simulation of how Gs- and Gq-coupled receptors affect the induction of long-term potentiation (LTP) and long-term depression (LTD) by filling and depleting AMPA receptor synaptic compartments. The upper row shows the effects on LTP; the lower row, the effect on LTD. Color conventions of compartments as in Figure 2. PeriIN: green; PeriOut: blue; Non-anchored: brown; postsynaptic density compartment: red; Synaptic conductance: black; actual data from Huang et al. (2012): gray circles. Left column **(A,B)** shows that Gs-GPCR stimulation (horizontally striped bar) fills up the PeriIN and PeriOut compartments, barely affecting the induction (gray vertical bar) of LTP **(A)**, but preventing the induction of LTD **(B)**. The middle column **(C,D)** shows that Gq-GPCR stimulation (vertically striped bar) depletes both the PeriIN and the PeriOut compartments, preventing the induction of LTP (C) without affecting the induction of LTD **(D)**. The right column **(E,F)** shows that co-stimulating Gs- and Gq-GPCRs fills up and depletes the PeriIN and the PeriOut compartments, respectively, allowing the induction of both LTP **(E)** and LTD **(F)**.

Subsequently, we checked whether the model accounts for the interactions between LTP/D and the neuromodulators. Indeed, the principal motivation for building the trafficking model was to account that while the activation of β- and α-adrenoreceptors individually prevent LTD and LTP, respectively, together they promote LTP and LTD. In the slice experiments, LTP and LTD were attempted at the end of the 10-min application of the neuromodulators (Huang et al., 2012). In the model, we used that timing sequence, and importantly we used the same values for parameters and constants optimized above in Figure 2. As shown in Figure 3, there was a clear concordance between the outcomes predicted by the model and the experimental data, both in the magnitude and time course of the changes in synaptic response. Like the activation of β- and α-adrenoreceptors in the slice, in the model Gs activation allows LTP and prevents LTD by saturating the perisynaptic compartments (Figures 3A,D), Gq activation allows LTD and prevents LTP by depleting the perisynaptic compartments (Figures 3B,E), and the coactivation of Gq and GS allows both LTP and LTD (Figures 3C,F).

**Figure 4:**
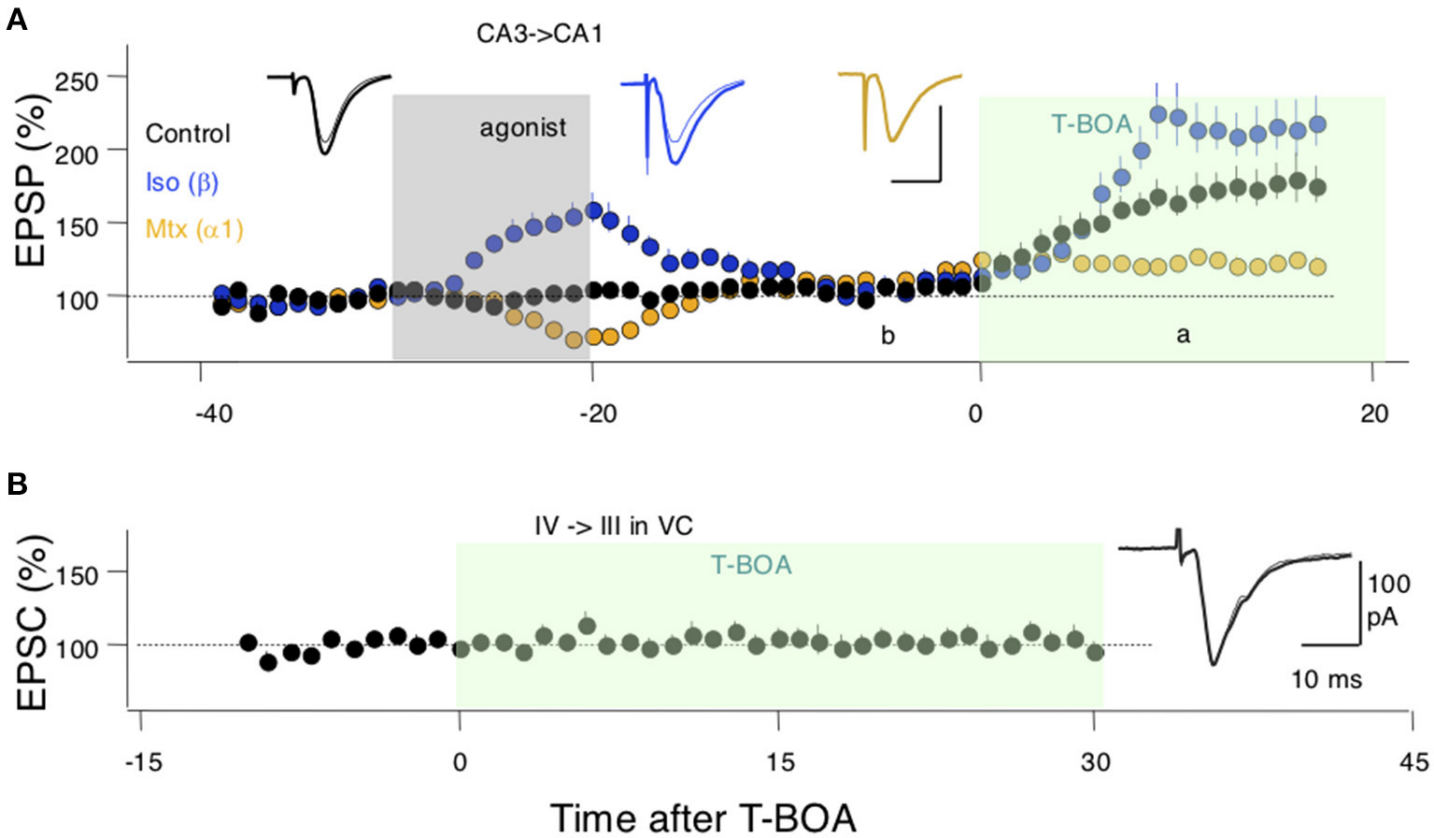
Evidence for an adrenergic modulation of a perisynaptic pool of AMPA receptors (AMPARs). **(A)** In SC-CA1 synapses of the hippocampus bath application of a glutamate uptake blocker (T-BOA: 10 μM. green box) enhances the synaptic responses (black symbols). Pretreating the slices with the agonist for β-adrenergic receptors (coupled to Gs) isoproterenol (Iso: 10 μM for 10 min) potentiates the effects of T-BOA. In contrast, pretreating with an agonist for the a1 adrenergic receptor (coupled to Gq) methoxamine (Mtx: 5 μM for 10 min) reduces the enhancement induced by T-BOA. **(B)** In the visual cortex, T-BOA does not affect synaptic responses suggesting that the pool of perisynaptic AMPARsis too small to be detected. Traces in **(A,B)** are averages of 10 consecutive responses recorded 10 min. before **(B)** and 10 min. after **(A)** the addition of T-BOA. Each symbol in **(A,B)** represents data normalized as % of pre-TBOA baseline and averaged over 1 min.

Finally, we sought experimental support for the idea, central to the model, that neuromodulators can affect perisynaptic AMPARs. To that end, we studied in hippocampal slices the CA3 → CA1 synapses, where perisynaptic AMPAR responses can be revealed and quantified by increasing glutamate spillover (Megill et al., 2015) via blocking glutamate uptake with the inhibitor TBOA (see methods). Bath-applied TBOA increases the amplitude and duration of the synaptic responses, reflecting the recruitment of extrasynaptic AMPARs. In the experimental design, we also exploited the previous observation that activation of β- and α-adrenoreceptors have lasting, “priming,” effects on LTP and LTD (Huang et al., 2012) that are consistent with lasting changes in the occupancy of the perisynaptic compartments. Thus, we asked how priming with noradrenergic agonists alters the effects of TBOA on synaptic responses recorded extracellularly as field potentials (FP) in CA1. As shown in Figure 4A, in the hippocampus, a 10-min pretreatment with the β-adrenergic agonist isoproterenol (Iso: 10 μM) increased the enhancements of the FP induced by bath-applied TBOA (10 μM); conversely, the α-adrenergic agonist methoxamine (Mtx:10 μM) reduced the TBOA-induced FP enhancement. An ANOVA test (*F*_2[2, 26]_ = 11.22; *p* = 0.0003) followed by Dunnett's *post-hoc* test confirmed the significance of the differences in the slopes measured 10–15 min after TBOA application. These results are consistent with a scenario in which β-and α-adrenoreceptors, respectively, increase and reduce the pool of extrasynaptic AMPARs. In the visual cortex, on the other hand, TBOA does not affect the synaptic response magnitude, responses collected in the −5 to 0 min interval prior to TBOA application, were comparable with those collected in the 25–30 min interval post TBOA application (paired *t*-test: *p* = 0.9616; Figure 4B). This might reflect a smaller capacity of the perisynaptic compartments or a larger distance from the synapse in the visual cortex (see section Discussion).

## Discussion

G-protein coupled receptors can facilitate and suppress LTP and LTD in a pull–push manner (Huang et al., 2012). To account for these opposite effects, we developed a simple model where neuromodulators modify the occupancy of two small and saturable perisynaptic compartments that limit the AMPAR traffic in and out of the synapse. This action at the expression level is sufficient to account for the suppression of LTP and LTD by Gs and Gq-coupled receptors, and their paradoxical synergy when simultaneously activated. Note that although GPCRs do affect Ca^2+^ signaling and the kinases and phosphatases involved in LTP/LTD induction (Pawlak et al., 2010; Tritsch and Sabatini, 2012; O'Dell et al., 2015; Meunier et al., 2017; Bari et al., 2020; Fernandez de Sevilla et al., 2021; Lutzu and Castillo, 2021), these actions are not required for the model to work. Indeed, for simplicity, they were not considered in this model. This contrasts with previous models explaining the facilitation of LTP and LTD induction in terms of changes in the kinase and phosphatase signaling pathways (Jedrzejewska-Szmek et al., 2017; Blackwell et al., 2019; Maki-Marttunen et al., 2020). We surmise that the two types of models, those focusing on the facilitation of induction via changes in kinases and phosphatases and this one focusing on the suppression of the expression *via* modulation AMPAR trafficking, are complementary and necessary for a comprehensive understanding of the neuromodulation of Hebbian plasticity.

Experimental evidence indicates that GPCRs can regulate LTP and LTD at the expression level independently of the well-documented facilitation of LTP and LTD induction. First, we have shown that the suppression of LTP and LTD by GPCRs is independent of changes in NMDAR activation and cell excitability (Huang et al., 2012). Second, stimulation of GPCRs and LTP/D induction can be dissociated in time. A brief GPCR stimulation epoch can prime the facilitation/suppression of LTP/D elicited even hours later (Tenorio et al., 2010; Huang et al., 2012; Hulme et al., 2012). GPCRs can also act retroactively after plasticity has been induced (Brzosko et al., 2015, 2017, 2019) or attempted (Yagishita et al., 2014; He et al., 2015; Fisher et al., 2017; Shindou et al., 2019). The trafficking model is well-suited to reproduce these temporal features of plasticity neuromodulation. GPCR-induced changes in perisynaptic compartment occupancy and its effect on LTP/D will tend to persist because AMPAR exchange is very slow at rest, following GPCR stimulation at rest. For example, a persistent saturation of these compartments after a brief and strong β-adrenergic stimulation would result in a lasting priming of LTP and lasting suppression of LTD (Huang et al., 2012). Conversely, a lasting depletion of these compartments after α-adrenergic or M1-muscarinic activation would result in the lasting priming of LTD and suppression of LTP (Huang et al., 2012). On the other hand, the retroactive actions of GPCRs, particularly the transformation of silent synaptic eligibility traces onto LTP and LTD, might reflect the combination of residual kinases/phosphatase activity and GPCR-induced changes in the occupancy of the perisynaptic compartments. In sum, the mechanistic dissociation of the induction (NMDAR activation, kinases/phosphatase activities) and the expression of plasticity (AMPAR modification and trafficking) provides a reasonable basis to account for defining temporal features of its neuromodulation.

As mentioned in the introduction, several distinct mechanisms for the expression of postsynaptic Hebbian plasticity have been identified, including lateral diffusion of AMPAR to and from the synapse, direct exchange with internal compartments, and changes in the unitary conductance of AMPAR; yet their relative contribution to LTP/D in different synapses remain unclear. Our model of pull–push neuromodulation, based on data from layer 4 → Layer 2/3 cortical synapses, is congruent with lateral diffusion only. How a pull–push neuromodulation could be implemented in the scenario of direct exo- and endocytosis or how GPCRs could affect changes in AMPAR unitary conductance in a pull–push manner remains to be explored, but it is not excluded as a possibility by our model. Our model also features the distinct assumption that AMPAR exchange to and from the PSD is channeled via two perisynaptic compartments whose occupancy, in turn, is controlled by GPCRs and constrains LTP and LTD. We arrived at this two compartments assumption due to the difficulty of fully modeling the simultaneous facilitation of LTP and LTD with a single compartment when Gs and Gq are coactivated. This is because facilitation of LTP by Gs-GPCRs would require saturating the single compartment whereas facilitation of LTD by Gq-GPCRs would require the depletion of that compartment. Simultaneous activation of Gs-GPCRs and Gq-GPCRs experimentally led to the facilitation of both LTP and LTD, and a parsimonious explanation involves the separation of these compartments. We did not explore other more complicated possibilities, for example, combining a single perisynaptic compartment with more complex schemes of trafficking signaling. The exact nature of these two hypothesized compartments and how GPCRs could control their occupancy remain open questions. A likely candidate mechanism for the occupancy and trafficking control could be the phosphorylation of a distinct constellation of sites in the AMPARs (Diering et al., 2016). In the few cases examined, different GPCRs phosphorylate different subsets of these sites (Hu et al., 2007; Seol et al., 2007; Huang et al., 2014), which in turn would be in tune with the notion of a “phosphorylation code” for AMPAR trafficking (Diering and Huganir, 2018).

Concerning the existence of two perisynaptic compartments, we note that the idea is consistent with the observation of a discrete and defined perisynaptic locus for endocytosis during LTD (Lu et al., 2007). Also consistent with the model are the results of Figure 4 showing that in the hippocampal slices GPCRs can control bidirectionally perisynaptic AMPARs revealed by blocking glutamate uptake. In visual cortical slices, blocking glutamate uptake did not reveal perisynaptic AMPARs, raising the intriguing possibility that the capacity of these perisynaptic compartments is smaller in cortical synapses than in hippocampal slices or that they are farther away from the synapse.

Finally, the trafficking model for the pull–push neuromodulation makes clear testable predictions. Indeed, a validating aspect of the model was that the values for constants and parameters obtained by fitting the LTP/LTD and Gs/Gq data independently predicted the effects of coactivating Gs and Gq on LTP/D. In Ca1, where the opposite modulation of LTP and LTD by individual GCPRs is well-documented (Katsuki et al., 1997; Mockett et al., 2007; Huang et al., 2012), the model predicts that the coactivation of Gs-GCPR and Gq-GCPR would promote both LTP at LTD. In addition, in CA1 and the visual cortex, the exposure to Gs-GCPR agonists can prime LTP facilitation and LTD suppression for an extended time (Huang et al., 2012). The model predicts that if priming reflects persistent saturation of perisomatic compartments then LTP facilitation and LTD suppression should decay at the same pace. In sum, we surmise that ours is a simple model that accounts for defining features of the pull–push neuromodulation of Hebbian plasticity and makes predictions that can be tested experimentally.

## Materials and Methods

### Slice Experiments

Experiments were performed according to the guidelines for the use of animals approved by the Ethics and Animal Care Committee of Universidad de Valparaíso (BEA064-2015) and the IACUC of Johns Hopkins University (MO14M404). Acute hippocampal or cortical slices were prepared from 1-month-old C57/BL6 mice as previously described (Huang et al., 2012; Ardiles et al., 2014). Briefly, each mouse was sacrificed by decapitation, following an overdose of isoflurane. Hippocampi were rapidly removed and sectioned into 350 μm slices using oxygenated ice-cold dissection buffer [composed of (in mm) 212.7 sucrose, 2.6 KCl, 1.23 NaH_2_PO_4_, 26 NaHCO_3_, 10 dextrose, 3 MgCl_2_, and 1 CaCl_2_] and recovered at room temperature in artificial CSF [ACSF; composed of (in mm) 124 NaCl, 5 KCl, 1.25 NaH_2_PO_4_, 26 NaHCO_3_, 10 dextrose, 1.5 MgCl_2_, and 2.5 CaCl_2_]. All recordings were done in a submersion recording chamber perfused with ACSF (29–30°C, 2 ml/min) bubbled with 95% O_2_/5% CO_2_. For FP recordings, synaptic responses were delivered through a bipolar glass stimulating electrode placed to activate the Schaffer collaterals with a 0.2-ms duration pulse (baseline stimulation at 0.0333 Hz) and recorded from the dendritic field of CA1. EPSCs in layer 2/3 pyramidal cells evoked by layer 4 stimulation were recorded as in Huang et al. (2012). Synaptic responses were digitized and stored online using Igor Pro software (WaveMetrics). To evaluate the neuromodulatory effect of Gs and Gq adrenoceptors, after 15 min of stable baseline, slices were superfused for 10 min with adrenergic agonists isoproterenol (Iso; 10 μm) and methoxamine (Mtx; 5 μm). Then 10 μm dl-threo-β-benzyloxyaspartic acid (TBOA, Tocris Biosciences), a competitive blocker of glutamate transporters, was used to induce spillover of glutamate and to reveal the activity of perisynaptic AMPARs. In these experiments, CA3 was cut away during dissection, and high divalents were added to the ACSF (4 mM MgCl_2_ and 4 mM CaCl_2_). Isolated AMPAR-mediated responses were evoked in the presence of 100 μM d,l-APV, and 2.5 μM gabazine. To prevent oxidation, isoproterenol and methoxamine were prepared freshly in ASCF containing sodium ascorbate (40 μM). FP slopes were measured and data are expressed as means ± SEM. All FP and EPSC data had a normal distribution as confirmed by D'Agostino–Pearson normality test. ANOVA and *t*-test were performed in Prism.

### Model

Three sets of equations describing the traffic of AMPARs between the PSD, PeriIN, PeriOut, UA, and Endo compartments and the occupancy of PSD, PeriIN, and PeriOut are described below. The set of equations represent three-time intervals, which are modulation, induction, and after induction. The dynamic equations are the same in the three-time intervals but differ in the constants used. The final values for the dynamics in one interval are used as the initial condition for the subsequent interval. The initial condition for the first interval is computed from the analytical solution of the dynamics reaching equilibrium at rest. The suffix A for each compartment denotes sites with anchored AMPAR, the suffix F denotes free sites; thus A plus F represents the total size of each compartment, which is constant in time during our simulations. Values of parameters and constants were chosen to fit the results of Figure 1 in Huang et al. (2012) and are indicated after the equations. Note that in that study, the induction protocols for Figure 1 were aimed at eliciting maximal plasticity; hence the rates of LTP and LTD, represented by Kin and PP, respectively are not affected by the neuromodulators. The ^*^model was written in Mathematica, and the code is available upon request.

#### Dynamical Equations

$$\begin{array}{l} \;{A^{\prime }}_{\;PeriIN}\left[t\right]=-k_{PeriINEndoM}*A_{PeriIN}\left[t\right]+k_{EntdoPeriINM}\\ \;*F_{PeriIN}\left[t\right]-k_{PeriINPsd}*Kin_{PeriIN}\left[t\right]\\ \;*A_{PeriIN}\left[t\right]*km2Kin/\left(km2Kin+A_{PeriIN}\left[t\right]\right)\\ \;*F_{Psd}\left[t\right]\\ \;{A^{\prime }}_{\;PeriOut}\left[t\right]=-k_{PeriOutEndoM}*A_{PeriOut}\left[t\right]+k_{EntdoPeriOutM}\\ \;*F_{PeriOut}\left[t\right]+k_{PsdPeriOut}*P_{\rho PeriOut}\left[t\right]\\ \;*A_{Psd}\left[t\right]*km2PP/\left(km2PP+A_{Psd}\left[t\right]\right)\\ \;*F_{PeriOut}\left[t\right]\\ \;{A^{\prime }}_{\;Psd}\left[t\right]=+k_{PeriINPsd}*Kin_{PeriIN}\left[t\right]*A_{PeriIn}\left[t\right]\\ \;*km2Kin/\left(km2Kin+A_{PeriIN}\left[t\right]\right)*F_{Psd}\left[t\right]\\ \;-k_{PsdPeriOut}*P_{\rho PeriOut}\left[t\right]*A_{Psd}\left[t\right]\\ \;*km2PP/\left(km2PP+A_{Psd}\left[t\right]\right)\;*F_{PeriOut}\left[t\right]\\ \;{A^{\prime }}_{\;UA}\left[t\right]=-kUA_{EndoM}*A_{UA}\left[t\right]+k_{EndoUAM}*A_{Endo}\left[t\right]\\ \;*F_{UA}\left[t\right],\\ Ki{n^{\prime }}_{\;PeriIN}\left[t\right]=-k_{Kin}*Kin_{PeriIN}\left[t\right]+k_{in},\\ P{\rho^{\prime }}_{\;PeriOut}\left[t\right]=-k_{P\rho }*P\rho_{PeriOut}\left[t\right]+k_{P\rho }, \end{array}$$

The first four equations represent the dynamics of the occupancy of the compartments. The next two equations represent normalized dynamics of the concentrations of kinases and phosphatases. These equations and parameters are identical during the modulation and induction. After induction, the equations are the same, but some of the parameters change. The parameters that change have an index M in the above equations and use the same parameter name without M (see the section Parameter Description) after the induction.

While the dynamical equations are kept general, allowing transitions between multiple compartments, it should be noted that several of these transition constants are zero in the model which reproduces well the observed data.

#### Initial Conditions

For the modulation period, the initial conditions start from the equilibrium value of the solution for the equations after induction.

For the induction period, the initial conditions are the end of the modulation period with an added step increase for the normalized kinases and phosphatases in the PeriIN and PeriOut compartments (KinPeriINInd, PpPeriOutInd).

For the after-induction period, the initial conditions are the end of the induction period.

#### Weight

The simulated weight represents the concentration of bound AMPAR in the PSD and the UA compartments. The weight is smoothed with an exponential kernel with time constant tau.

#### Parameters

It should be noted that given the complexity of the model compared with the number of experimental observations an automatic parameter tuning could not be performed. Therefore, it is important to view these parameters as a possible explanation for the observed data, and not necessarily the only explanation for the observed data. While this is a significant limitation of the current model, we believe the model offers an important conceptual description of the phenomenology, and more precise estimates of the large number of parameters involved would require a vast array of measurements of the biochemical processes involved.

The modulation period lasts 9 min, induction 2, and the total simulation time is 50 min. The unit of time used is minutes.

The total sizes of different compartments are normalized relative to the size of the PSD compartment (TPsd = 1). For the recorded data, there was no clear need to differentiate their respective sizes, and they were all simulated to be equal and quite a bit smaller than the PSD (TAU = TPeriIn = TPeriOut = 0.2).

The rare constants are normalized such that the free AMPAR concentration is 1. While the equations are written in general for the movement of the AMPAR between compartments, the constants characterizing the movement from PeriIn to PSD and PSD to PeriOut are the only non-zero constants (kPeriINPsd = 0.001, kPsdPeriOut = 0.004). Both are very slow, requiring hours to reach equilibrium occupancy (i.e., for the synapse to revert to its background state), and the out rate is higher, resulting in an equilibrium state which is biased toward the PSD anchoring, options being mostly free.

Following LTP or LTD induction, these rates change significantly. They change by a multiplicative factor (KinPeriIN and PpPeriOut), which is time-dependent and abstractly approximates the effects of kinases and phosphatases. These multiplicative factors are computed in the last two dynamic equations. Their values are defined to be normalized to their resting values (the equilibrium solution is defined to be 1 for these variables). Following an LTP induction, KinPeriIn is increased by a factor of KinPeriINInd = 1,000 and reverts to equilibrium with a time constant of kKin = 1/(4 min), while PpPeriOut increases by a factor of PpPeriOutInd = 400 and reverts back to equilibrium with a time constant of kPp = 1/(10 min). The movement to PeriIN and PeriOut compartments is subject to saturation, with saturation half-activations km2Kin = 0.1 and km2Pp = 0.1, which were kept fix at half the size of the Peri compartments.

Lastly, without modulation, the rates from and to the Peri compartments to the Endo(somal) compartment are fixed at 1/(50 min) and from UA to and from Endo at 1/(20 min). Application of beta-agonists leads to an increase of movement from Endo PeriIn by a factor of 100, to PeriOut by a factor of 10, and to UA by a factor of 4.5. Application of alpha agonists leads to an increase in movement to Endo from PeriOut by a factor of 100, from PeriIn by a factor of 10, and from UA by a factor of 2.

## Data Availability Statement

The raw data supporting the conclusions of this article will be made available by the authors, without undue reservation.

## Ethics Statement

The animal study was reviewed and approved by Ethics and Animal Care Committee of Universidad de Valparaíso (BEA064-2015).

## Author Contributions

SM and AK developed the model and wrote the manuscript. SM implemented the model. AA, AP, and AK designed the slice experiments. AA conducted the experiments. All authors contributed to the article and approved the submitted version.

## Conflict of Interest

The authors declare that the research was conducted in the absence of any commercial or financial relationships that could be construed as a potential conflict of interest.

## Publisher's Note

All claims expressed in this article are solely those of the authors and do not necessarily represent those of their affiliated organizations, or those of the publisher, the editors and the reviewers. Any product that may be evaluated in this article, or claim that may be made by its manufacturer, is not guaranteed or endorsed by the publisher.
